# “Get Me Off This Medication!”: A Comparison Between Gastroenterology and Primary Care Regarding Patient’s Perceptions of Proton Pump Inhibitor Therapy

**DOI:** 10.7759/cureus.11158

**Published:** 2020-10-25

**Authors:** Raja Samir Khan, Yousaf Hadi, Swapna Gayam

**Affiliations:** 1 Internal Medicine, West Virginia University School of Medicine, Morgantown, USA; 2 Digestive Diseases, West Virginia University School of Medicine, Morgantown, USA

**Keywords:** proton pump inhibitor, perspectives, discontinuation, adverse effects

## Abstract

Background

Not much is known about patient perceptions regarding proton pump inhibitor (PPI) de-escalation. We sought to determine the knowledge of adverse effects (AEs) and willingness to de-escalate therapy among patients presenting to primary care and subspecialty clinics.

Methods

We conducted an anonymous survey of patients presenting to family medicine, internal medicine, and gastroenterology clinics who use PPIs. Survey topics included awareness of and concern for AEs of PPIs, and willingness to de-escalate PPI therapy.

Results

The sample comprised 206 participants presenting to the gastroenterology (29.8%), internal medicine (32.2%), and family medicine clinics (38%). Of the participants, 16% were “extremely concerned” about AEs and 28.2% reported attempting to stop PPIs by themselves in the past. Many patients (54.9%) reported that providers had not discussed AEs before initiation. Patients visiting digestive disease clinics were no more likely to report discussions on AEs and de-escalation or discontinuation attempts compared to primary care patients (p-values > 0.05). On logistic regression analysis, concern for AEs and counseling regarding PPI discontinuation were found to be significantly associated with attempts to discontinue PPI.

Conclusions

Although many patients on PPIs are concerned about AEs, a low number of patients reported provider-initiated discussions on AEs of PPI at initiation.

## Introduction

Proton pump inhibitors (PPIs) have been documented extensively in the literature to be among the most commonly prescribed drug classes in the United States [1]. A growing number of studies have noted the association of PPIs with adverse effects such as *Clostridium difficile* infection [2], fractures [3], and acute and chronic kidney disease [4]. There has also been much discussion regarding the clinical significance of such side effects and their association with PPIs [5], with emphasis on potential discontinuation or dose reduction of these medications [6]. Recently, PPI de-escalation initiatives have been launched at various institutions [7,8]. PPIs are available over the counter and are prescribed by both primary care and specialist gastroenterologists. Per a national survey that sought to evaluate the association between concerns regarding PPI-related side effects and prior attempts to stop such medications, it was noted that concern regarding the side effects of PPIs was common and strongly associated with attempts to stop taking the medication regardless of a prescriber’s recommendation [9]. Despite the attention that PPIs and their adverse effects have garnered in the literature currently, little is known about how such adverse effects are perceived by patients presenting to different subspecialties and primary care. In this study, we sought to determine the difference in knowledge of such adverse effects and willingness to de-escalate or discontinue PPI therapy between patients presenting to primary care and those presenting to subspecialty gastroenterology clinics.

## Materials and methods

We conducted a survey at clinics operated by West Virginia University. Before commencement, the study was reviewed and approved by our institutional review board. Patients more than 18 years of age presenting to family medicine, internal medicine, and gastroenterology clinics at West Virginia University were presented the study advertisement and consent at the check-in desk; they could self-administer the survey if they wished to enroll. Patients were asked to complete the survey only if they were taking a PPI. A list of generic and trade names of PPIs was included in the cover letter. They then deposited the survey in a drop box placed at the check-in desk after completing the survey.

The questionnaire was adapted and modified from a previously tested questionnaire developed by the University of Michigan [9]. The survey remained active for one month. No incentive was offered for completing the survey.

The questionnaire included multiple-choice questions on baseline demographics, use of PPIs, knowledge and perceptions regarding adverse effects of PPIs, indications for PPIs, and previous attempts and perceptions regarding PPI discontinuation.

Patients were considered at high risk of gastrointestinal (GI) bleeding if they were on dual antiplatelet therapy, if they were using anticoagulants or steroids along with anti-platelet agents or non-steroidal anti-inflammatory drugs (NSAIDs), or if they were taking antiplatelet agents or NSAIDs in the setting of a history of peptic ulcer disease. A multivariable logistic regression model was applied with “any attempt at discontinuation due to adverse effect” as the dependent variable, and age, gender, specialist vs primary care clinic, whether the patient was told to discontinue PPI by their provider in the past, and the level of concern for adverse effect endorsed by the patient as covariates. All p-values less than 0.05 were considered significant. Data analysis was completed using the software SPSS Version 20 (IBM Corp., Armonk, NY, USA).

## Results

Population characteristics

A total of 206 survey responses were received. Most participants were Caucasian (88.8%), and the sample comprised a majority of females (59.7%). The mean age of participants was 54.92 years (standard deviation: 15.74). 

Of these patients, 78 patients were enrolled from family medicine clinics (38%), whereas 66 (32.2%) and 61 (29.8%) patients were presenting to internal medicine and gastroenterology clinics, respectively. Omeprazole was the most common PPI among the study population (38.8%) followed by pantoprazole (25.7%). 

Most patients were taking PPI with a diagnosis of gastroesophageal reflux disease (GERD) (87.4%). Most patients reported that they were established with a primary care physician (PCP) (93.2%) and believed that the PPI had been prescribed by their PCP (61.7%); 44 patients attributed the PPI prescription to a GI specialist (21.4%). Twelve patients did not recall the provider who prescribed the PPI (5.8%). 

Over-the-counter PPI use was reported by 18.4% of participants. Characteristics of the study population are detailed in Table 1.

**Table 1:** Characteristics of the study population PPI, proton pump inhibitor; GERD, gastroesophageal reflux disease; GI, gastrointestinal

Variable	N (%)
Gender	
Male	81 (39.3%)
Female	123 (59.7%)
Transgender	2 (1%)
Race	
Caucasian	123 (88.8%)
African American	5 (2.4%)
Asian	3 (1.5%)
Native American	3 (1.5%)
Hispanic	2 (1.0%)
Other	3 (2.5%)
Prefer not to say	7 (3.4%)
Visiting clinic	
Family medicine	78 (38%)
Internal medicine	66 (32.2%)
Digestive diseases	61 (29.8%)
Prescriber of PPI	
Primary care provider	127 (61.7%)
Digestive diseases	44 (21.4%)
Pulmonology	2 (1.0%)
Ear, nose, and throat	10 (4.9%)
Others	15 (7.3%)
Over the counter	8 (3.9%)
Taking PPI for GERD	
Yes	180 (87.4%)
No	26 (12.6%)
Non-GERD indications for PPI	
Peptic ulcer disease	38 (18.4%)
Esophagitis	44 (21.4%)
Barrett’s esophagus	9 (4.4%)
History of GI bleed	9 (4.4%)
Helicobacter pylori	17 (8.3%)
High risk of GI bleeding	
Yes	79 (38.3%)
No	127 (61.7%)

Knowledge and perspectives of adverse effects

Of the sample, 16% and 28.6% of the participants reported being extremely concerned and somewhat concerned about adverse effects of PPIs, respectively. However, a majority of patients reported being “not at all” or “slightly” familiar with reports of adverse effects (36.4% and 27.7%, respectively).

A minority of patients (9.2%) reported having ever experiencing an adverse effect with PPIs. Of those who had been diagnosed with an adverse effect of PPI, only 3.8% reported that their provider had discussed discontinuation of PPI; 58.3% patients were “very comfortable” about discussing PPI discontinuation with their provider.

A majority of patients reported that their prescribing practitioner had not discussed adverse effects when prescribing the PPI (54.9%), whereas 27.7% patients reported that could not recall any such conversation.

Impact of PPI on quality of life

Most patients (59.2%) either reported no GERD symptoms or reported their severity as just noticeable, and of the total sample, 86.8% of participants reported moderate-to-complete resolution of GERD symptoms with PPI use. A majority of patients (72.3%) reported that they could probably or definitely not manage their symptoms without PPIs. Overall, 76.7% of patients attributed PPIs to have moderate-to-high effect on their quality of life.

Perspectives and practices regarding PPI discontinuation

Of the participants, 28.2% reported that they had themselves tried to discontinue PPIs and 30.6% reported attempts at reducing the dose of PPIs by themselves in the past. 

A minority of patients (22.8%) reported that their health care practitioner had attempted reducing their PPI dose or attempted to discontinue their PPI completely (10.7%). 

Overall, a majority of patients rated their willingness to reduce or stop PPI favorably, with 38.8% and 22.3% of participants reporting that they were “somewhat willing” and “very willing” to discontinue PPI, respectively. Perspectives of patients regarding PPI discontinuation are detailed in Table 2.

**Table 2:** Perspectives of patients regarding PPI adverse effects and discontinuation PPI, proton pump inhibitor; HCP, health care provider; GERD, gastroesophageal reflux disease

Variable	N (%)
Concern about adverse effects	
Not at all concerned	45 (21.8%)
Slightly concerned	69 (33.5%)
Somewhat concerned	59 (28.6%)
Extremely concerned	33 (16.0%)
Attempted to stop taking PPI	
Yes	58 (28.2%)
No	148 (71.8%)
Attempted to reduce PPI dosage	
Yes	63 (30.6%)
No	143 (69.4%)
HCP discussion regarding adverse effects of PPIs	
Yes	36 (17.5%)
No	113 (54.9%)
Cannot recall	57 (27.7%)
HCP discussion regarding dose reduction of PPIs	
Yes	47 (22.8%)
No	140 (68.0%)
Cannot recall	19 (9.2%)
HCP discussion regarding discontinuation of PPIs	
Yes	22 (10.7%)
No	159 (77.2%)
Cannot recall	25 (12.1%)
Willingness to reduce dosage of PPI	
Very unwilling	22 (10.7%)
Somewhat unwilling	38 (18.4%)
Somewhat willing	80 (38.8%)
Very willing	66 (32.0%)
Willingness to stop taking PPI	
Very unwilling	31 (15.0%)
Somewhat unwilling	49 (23.8%)
Somewhat willing	80 (38.8%)
Very willing	46 (22.3%)
Willingness to stop taking PPI if able to go back on current dose if needed	
Very unwilling	27 (13.1%)
Somewhat unwilling	26 (12.6%)
Somewhat willing	77 (37.4%)
Very willing	76 (36.9%)
Willingness to stop taking PPI and take less powerful GERD medication	
Very unwilling	29 (14.1%)
Somewhat unwilling	33 (16.0%)
Somewhat willing	76 (36.9%)
Very willing	68 (33.0%)
Willingness to stop taking PPI if tapered off slowly	
Very unwilling	20 (9.7%)
Somewhat unwilling	20 (9.7%)
Somewhat willing	92 (44.7%)
Very willing	74 (35.9%)

Regarding strategies to discontinue PPI, participants rated all strategies favorably. They were “somewhat willing” and “very willing” to attempt discontinuation if they were asked to slowly asked to reduce dose (80.6%), or with alternative strategies including “stop and replace with H2 blocker” (69.9%) or “stop and be allowed to go back on current dosage if discontinuation fails” (74.3%).

Factors associated with attempts at discontinuation

A logistic regression model was applied incorporating age, gender, specialist vs primary care clinic, whether the patient was told to discontinue PPI by their provider in the past, and the level of concern for adverse effect endorsed by the patient. Results of the regression are detailed in Table 3. Compared to patients who were “not at all concerned” about adverse effects, patients who were “somewhat” or “extremely” concerned about adverse effects of PPIs were significantly more likely to have attempted PPI discontinuation in the past. Patients who had been told to discontinue PPIs by providers were also more likely to have attempted discontinuation. There was no difference regarding attempts at discontinuation between patients who were presenting to a specialist and those presenting to primary care clinics.

**Table 3:** Logistic regression analysis for factors associated with attempts at discontinuation of proton pump inhibitor therapy HCP, health care provider

Variable	Odds Ratio	95% Confidence Interval	p-Value
Age	1.002	0.978-1.026	0.882
Gender			
Male	Reference		
Female	0.994	0.479-2.065	0.988
Transgender	3.789	0/197-73.060	0.378
Concern for adverse effects			
Not at all concerned	Reference		
Slightly concerned	2.347	0.678-8.131	0.178
Somewhat concerned	6.015	1.760-20.562	0.004
Extremely concerned	13.764	3.752-50.494	0.000
High risk of bleeding			
No	Reference		
Yes	1.407	0.668-2.964	0.370
HCP recommended discontinuation			
No	Reference		
Yes	9.129	3.106-26.835	0.000
Clinic specialty			
Primary care	Reference		
Digestive disease	0.784	0.356-1.725	0.545

## Discussion

Based on our survey, it is noted that 44.6% of the sample population endorse concerns about the adverse effects of using PPIs for GERD and 30.6% endorse having tried to reduce the dosage or stop PPIs in the past without the recommendation of their physician (28.2%). Per literature, it is noted that patients at high risk of upper gastrointestinal bleeding due to pharmacological and clinical risk factors benefit from ongoing PPI usage [5,10]. The national survey conducted recently noted that most patients using PPIs for GERD endorse concerns about adverse effects with strong association with prior attempts to stop these medications, and most patients who attempted to stop PPIs also did so without an official recommendation from their physician [9]. These findings are consistent with our survey, and when taken together, they suggest that PPI de-escalation attempts are endorsed by a minority of patients, and even in that population, most de-escalation attempts are patient-led. Provider-led attempts at de-escalation were only endorsed by 10% of the population studied in our cohort. Furthermore, the finding that many patients attempt discontinuation on their own also raises some concern. This is especially important, considering that there was no difference in prior attempts to stop PPIs in patients who were at a high risk of GI bleeding compared to those who were not.

The reasoning behind attempts to discontinue PPI therapy without provider recommendation requires further study and exploration. In our study, 54.9% of the sample population endorsed not having a discussion with their provider regarding the risks and benefits of PPI therapy, and a previous study noted that 24% patients endorsed having a discussion with their provider [9].

Patients should always be advised to talk with their providers regarding the potential adverse effects of the medications they are taking before attempting to make any medication changes, even those involving medications that are available over the counter. Providers should also assume a more responsible role in discussing the side effects of the PPIs their patients are currently taking, the intended duration of treatment, which, per literature, is a practice that is routinely overlooked [11], and the potential risks of stopping PPIs in patients who meet clinical criteria for benefit with continuation, as well as engage in shared decision-making with the patients on long-term PPI therapy without an appropriate indication in order to ensure that the decision to discontinue or de-escalate the medication is an informed decision [12,13].

Our survey does find that most patients presenting to clinics are willing to de-escalate PPIs, even if a large proportion of these patients attribute PPIs to a significant improvement in their quality of life. Many patients believed that they could probably or definitely not control their GERD symptoms without PPIs, but they still expressed willingness to de-escalate PPI therapy. Therefore, it appears that the major barrier to PPI de-escalation may lie at the level of the provider. This is consistent with recent literature, where electronic health record based interventions directed at physicians have led to a decrease in PPI prescription [14].

The strengths of our study include recruitment of a diverse population presenting to both primary care and specialist clinics. Previous data have focused on internet-based surveys, which may appeal certain demographics of survey takers. However, our sample may under-represent over-the counter PPI users due to the nature of our study.

## Conclusions

Our study thus complements previous data and in conjunction draws the important conclusion that most PPI de-escalation attempts in the United States remain patient-led, with only a small minority of patients recalling risk-benefit discussions with their providers, and that provider recommendation was associated with a high likelihood of de-escalation attempt. Therefore, we conclude that more informed conversations with patients by providers may lead to de-escalations in patients that need it and potentially reduce inappropriate self-initiated de-escalation by patients.
